# Faster evolving *Drosophila* paralogs lose expression rate and ubiquity and accumulate more non-synonymous SNPs

**DOI:** 10.1186/1745-6150-9-2

**Published:** 2014-01-17

**Authors:** Lev Y Yampolsky, Michael A Bouzinier

**Affiliations:** 1 Department of Biological sciences, East Tennessee State University, Johnson City, TN 37614, USA; 2 InterSystems Corporation, One Memorial Drive, Cambridge, MA 02142, USA

**Keywords:** Gene duplication, Pseudogenization, *Drosophila*, Substitution rate, Gene expression, Polymorphism

## Abstract

**Background:**

Duplicated genes can indefinately persist in genomes if either both copies retain the original function due to dosage benefit (gene conservation), or one of the copies assumes a novel function (neofunctionalization), or both copies become required to perform the function previously accomplished by a single copy (subfunctionalization), or through a combination of these mechanisms. Different models of duplication retention imply different predictions about substitution rates in the coding portion of paralogs and about asymmetry of these rates.

**Results:**

We analyse sequence evolution asymmetry in paralogs present in 12 *Drosophila* genomes using the nearest non-duplicated orthologous outgroup as a reference. Those paralogs present in *D. melanogaster* are analysed in conjunction with the asymmetry of expression rate and ubiquity and of segregating non-synonymous polymorphisms in the same paralogs. Paralogs accumulate substitutions, on average, faster than their nearest singleton orthologs. The distribution of paralogs’ substitution rate asymmetry is overdispersed relative to that of orthologous clades, containing disproportionally more unusually symmetric and unusually asymmetric clades. We show that paralogs are more asymmetric in: a) clades orthologous to highly constrained singleton genes; b) genes with high expression level; c) genes with ubiquitous expression and d) non-tandem duplications. We further demonstrate that, in each asymmetrically evolving pair of paralogs, the faster evolving member of the pair tends to have lower average expression rate, lower expression uniformity and higher frequency of non-synonymous SNPs than its slower evolving counterpart.

**Conclusions:**

Our findings are consistent with the hypothesis that many duplications in *Drosophila* are retained despite stabilising selection being more relaxed in one of the paralogs than in the other, suggesting a widespread unfinished pseudogenization. This phenomenon is likely to make detection of neo- and subfunctionalization signatures difficult, as these models of duplication retention also predict asymmetries in substitution rates and expression profiles.

**Reviewers:**

This article has been reviewed by Dr. Jia Zeng (nominated by Dr. I. King Jordan), Dr. Fyodor Kondrashov and Dr. Yuri Wolf.

## Background

Retained gene duplication is probably the most important mechanism of evolution of genes and gene functions [1-7]. While many duplications, including the majority of genes resulting from whole-genome duplications, are rapidly lost by pseudogenization, many are retained. Paralogs can be retained when both copies continue to perform the original function due to dosage benefit, i.e., by gene conservation [1,6,8], also described as “more of the same” mechanism [5]. Alternatively, one of the copies may undergo neofunctionalization: assuming a novel function due to relaxation of stabilising selection in that copy [1,9,10]. Finally, subfunctionalization may occur where both copies become necessary to perform the function previously accomplished by a single copy. This may happen either by fixation of hypomorphic alleles in both copies or due to differential loss of regulatory elements [3,7,11,12]. Each of these duplication retention models implies different predictions about relaxation of stabilising selection in neither, one, or both paralogs [6,7]. During pseudogenization and neofunctionalization selective constraints are relaxed in one copy but not the other; such pairs of paralogs may be expected to evolve in an unusually asymmetric manner relative to the null expectation of equal rates in both paralogous clades: one paralog accumulates more mutations than the other and that difference is too large to be explained by binomial process of incurring random mutations. Conversely, paralogs retained by gene conservation or balanced degradation type of subfunctionalization may be hypothesised to accumulate changes in an unusually symmetric manner, i.e. with rates more similar than one might expect from the random null expectation, something that can be much harder to detect and test. Such symmetry may be further explained by the action of gene conversion or unequal crossing over homogenizing paralogous copies [13].

There are several important caveats that make the analysis of rates’ asymmetries in paralogs difficult and prone to false negatives. Firstly, both neofunctionalization and subfunctionalization often occur by loss or gain of entire protein domains [14,15] or by fixation of alternative splicing modes [16], neither of which would be detected by a comparison of aligned amino acid sequences. Likewise, both neofunctionalization and subfunctionalization may occur through a single or few amino acid substitutions at functionally important sites. A gene-wide analysis of rates and asymmetries will never have sufficient resolution to detect these few critically important substitutions. Finally, different modes of duplication retention may be difficult to distinguish, as they may not be mutually exclusive. For example, neofunctionalization may be accompanied by asymmetric expression profiles between the two paralogs, such as in the case of pancreatic ribonuclease in leaf-eating monkeys [17,18] or β-catenin/*armadillo* paralogs in insects [19], although it is not clear if faster-evolving paralogs generally tend to also acquire a more specialized expression. Moreover, there is compelling evidence that paralogs may evolve through a combination of subfunctionalization and neofunctionalization [20,21] with subfunctionalization often prevailing in the initial phases of duplicated genes evolution, setting the stage for further neofunctionalization [20]. Furthermore, it is particularly difficult to unequivocally distinguish between neofunctionalization and unfinished (or incomplete) pseudogenization, as both mechanisms predict identical signatures: relaxed selective constraint and high asymmetry in paralogs.

For the reasons outlined above, it is difficult to distinguish between different modes of duplication retention by the analysis of sequence evolution alone. Yet, one might attempt to detect genome-wide patterns of several of these modes by a joint analysis of rates and asymmetry of sequence evolution, gene expression and polymorphisms segregating in extant copies of duplicated genes.

Paralogous genes often demonstrate a faster divergence than their singleton counterparts [6,22-24] and their divergence can also be more asymmetric that that of orthologous clades [24-29]. However, genome-wide comparison of rates and asymmetries of sequence evolution between paralogs and singletons is difficult owing to the lack of a properly matched control: conserved gene families are also more likely to retain duplications [30,31], (but compare to Ref. [32]), thus creating an apparent decrease in evolution rate in paralogs than in singletons in a genome-wide comparison [33]. One approach to eliminate this confounding is to compare paralogs to their pre-duplication sister singleton orthologs [9,10,13,26], which requires sequence data analysed on a detailed phylogenetic context encompassing a number of closely related genomes, and thus was, until recently, limited to unicellular organisms. Here we report the analysis of rates and asymmetries of sequence and expression evolution in paralogous genes present in twelve *Drosophila* genomes [34] juxtaposed to those in their nearest singleton outgroup, a strategy recently employed for the analysis of duplicated genes in pairs of species of yeast [26], *Drosophila*[10] and rodents [9], but, to the best of our knowledge, has not been used on data encompassing entire phylogenies. Using the 12 *Drosophila* genomes and detailed phylogeny information available, we identify pairs of paralogs whose divergence is significantly more asymmetric than one would expect based on random distribution of substitutions across paralogous clades. We then focus on a subset of duplication with both copies present in the *D. melanogaster* genome in order to analyse the asymmetry of their sequence evolution in conjunction with gene expression data and the data on frequencies of SNPs mapped to these paralogs. Such analysis provides additional information about intensity of stabilising selection in each duplicated copy.

## Methods

### Data provenance and substitutions database

We analysed amino acid substitutions in a nearly complete set of multiple amino acid sequence alignments from 12 completely sequenced *Drosophila* genomes [34,35]. Protein alignments and corresponding phylogenies reconciled by NOTUNG algorithm [36] were acquired from Dfam database [35] (http://www.indiana.edu/~hahnlab/fly/DfamDB/drosophila_frb.html). To exclude areas of low alignment quality, indels of more than 1 amino acid long and 5 flanking sites straddling each such indel were excluded from consideration. The resulting database of amino acid substitution in its current form includes 8,212,684 amino acid substitutions occurring in 12 drosophilid genomes spanning 11567 gene families in which at least 6 species are represented. Using NOTUNG reconciliation every node in each tree was assigned to one of two types: speciation or duplication and every edge and every leaf (extant gene) to one of three gene types – paralogs, true singletons and remaining paralog from a lost duplication. This allowed us to classify each substitution according to its phylogenetic mapping to either singleton or duplicated gene. Details of the database structure as well as the preliminary analysis have been reported earlier [24]; the database and code necessary to generate it from alignments data are available from http://sourceforge.net/projects/acidminer.

### Phylogenetic analysis

For each site containing one or more substitutions, the ancestral state has been reconstructed for each node of the tree, either by parsimony (when a non-ambiguous reconstruction was possible; about 1/4 of all divergent sites) or, when multiple equally parsimonious reconstructions were possible, by means of assigning posterior probabilities of each of these reconstructions based upon the matrices of pair-wise frequencies of amino acid substitutions. These matrices were constructed on the basis of the non-ambiguous substitutions for each of 10 binned classes of phylogeny depth uniformly sampling the entire depth of drosophilid phylogeny in order to account for the possibility that different substitutions have different likelihood on different evolutionary times. Thus, each substitution was assigned a posterior probability (1 in case of a single most parsimonious reconstruction). The sum of posterior probabilities of all alternative equally parsimonious reconstructions was 1 for each substitution.

To calculate the rate of substitutions in a given clade, the sum of all posterior probabilities assigned to hypothesised substitutions occurring between the clade’s common ancestor and any of the leaves was calculated and weighted by the sum of alignment lengths between the reconstructed ancestral state and each known extant leaf (after filtering out indels >1 amino acid long and flanking regions, see above). Only paralogous leaves were used for this calculations, i.e., if one of the paralogs was subsequently lost in one or more species in one of the two paralogous clades, the remaining paralog was not used. This approach resulted in a central estimate of K_a_ for a given clade reflecting the rate of evolution of this paralog in all species. In order to analyse a relative rate of substitution, this estimate was normalized by the depth of the ancestral node known from the 12-species phylogeny [34], measured as the frequency of synonymous substitutions per 4-fold degenerate site. This normalized rate will be referred to as relK_a_. Of course, the depth of a duplication node is not known with any greater precision than the depths of the preceding and following duplication nodes. Thus, the depth of the nearest preceding speciation event was used as the upper-bound estimate of the node depth for duplication nodes. The same sums of posterior probabilities were used as weights to calculate clade-specific averages of absolute change in polarity and exchangeabilities (see below).

**Figure 1:**
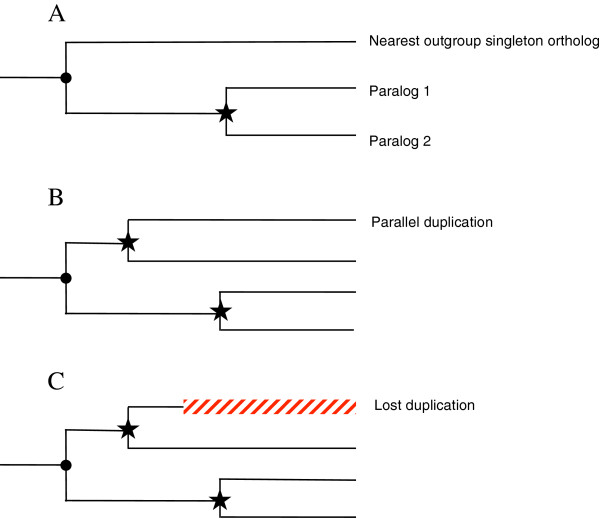
**Topologies of trees with duplications. A**: Phylogeny topology used for the placement of substitutions on the paralogous branches. **B** and **C**: discarded topologies (duplication followed by a loss of one of paralogs and nested duplication). Dot: speciation event; star: duplication event; green: used duplications, red: discarded duplications.

In order to compare the rate of substitutions in each paralog to that in their outgroup singleton ortholog, we calculated substitution rates by comparing the extant sequences of singletons and paralogs to the ancestral state reconstruction of the speciation node directly preceding a duplication node (Figure 1). This approach has the following advantages: i) it allows matching a pair of paralogs to its phylogenetically closest gene that remained a singleton and ii) it allows detecting substitutions that occurred in both paralogs due to concerted evolution [37]. Such duplications would not be detectable if paralog sequences were compared to the ancestral state reconstructed at the duplication node.

In this study we did not use the information about mapping substitutions to individual edges of the phylogeny. Yet, we predict that if such information can be reliably obtained and concerted evolution is taking place, one would observe an unusually high rate of substitutions in the edge directly preceding a duplication node, thus creating spurious evidence of accelerated evolution in genes immediately prior to duplication, or, conversely, making it more difficult to observe the expected post-duplication rates acceleration (cf. [9]).

The disadvantage of this approach is that the paralogs had a shorter (and unknown) time to diverge than the time of divergence of the singleton outgroup from either paralog. As a result, we are bound to overestimate the asymmetry of the paralogs’ evolution rate relative to that between the ortholog and its sister clade containing the duplication. This is, therefore, a conservative approach for both main purposes of this paper: demonstrating that the mean rate of evolution of paralogs is higher than that of the orthologous singleton and that the asymmetry of paralogs’ evolution is higher than that between the orthologous singleton and the clade with the duplication.

Three filters were employed to select suitable duplications for the analysis (Figure 1). Firstly, the requirement of the singleton outgroup excluded duplications present in the ancestor of the 12 *Drosophila* species. Secondly, the same requirement implied the selection of the outermost duplication in case of nested duplications (i.e., the ancestral node occurs in a singleton, Figure 1B); this also allowed us to avoid non-independent data points stemming from nested duplication. Finally, a duplication was also filtered out if its nearest outgroup was predicted to be a single remaining paralogs after a loss duplication (Figure 1C). Thus, only duplications that are directly preceded by a speciation event in a singleton gene were selected for this analysis. Yet, it was possible to have more than one duplication per family if they occurred in (were phylogenetically reconstructed to) parallel orthologous clades or if they occurred in a previously duplicated clade, which later reverted to the singleton status due to duplication loss. These filters resulted in 1726 duplications in 1470 families retained for the analysis (Table 1).

**Table 1:** **Summary of data used (See Additional file**3**: Table S1 and Additional file**4**: Table S2 for whole dataset)**

**Dataset**		**Number of gene families**	**Number of duplications**	**Number of pairs of paralogs**
Whole phylogeny		1470	1726	3398
*D. melanogaster*	Total	175	175	314
	Has expression data	162	162	276
	Has SNP data			
	(>2 SNPs per gene)	141	141	239

The number of paralog pairs is higher than the number of duplications because of nested duplications (only the outermost, i.e., oldest duplication was included in the analysis, but substitution rates observed for all nested paralogs were averaged to obtain the mean estimate).

### Expression and polymorphism data

A much smaller set of pairs of paralogs (175 duplications in 175 families) in which both members are present in the *D. melanogaster* genome was created to analyse the asymmetry of paralogs’ evolution in conjunction with gene expression, chromosomal location, and extant polymorphism data (Table 1). For each such paralog, gene ontology and chromosomal location data were obtained from FlyBase [38], using FBgn ID as the merging index and allowing for synonyms. For FBgn IDs present in Dfam data, for which no Flybase entry was found, Flybase’s ID Convertor tool (http://flybase.org/static_pages/downloads/IDConv.html) was used to establish the correspondence between the “Secondary ID” present in Dfam and “Current ID” used in the current version of FlyBase, and the data found for either Current ID or Secondary ID was used for a given paralog. Likewise, both the Current ID or Secondary ID were used to match paralogs with the expression data Fly Atlas [39] and to SNP data obtained from DGRP resequencing project [40] (annotation_5_46; http://dgrp.gnets.ncsu.edu/webdata). In a number of cases FBgn IDs assigned to paralogs in the Dfam database map to the same FlyBase Current ID (and thus the same record in the expression data), possibly indicating that previously described tandem paralogs have been subsequently recognized as isoforms of the same gene. Such cases were removed form the dataset, reducing the number of duplications in the analysis to 162. Only paralogs in which there were at least three synonymous and at least three non-synonymous SNPs were retained for the polymorphism analysis, reducing the number of analysed duplications to 141 (Table 1). While potentially creating a bias against genes with low levels of polymorphism (there were 50 pairs of paralogs eliminated), this filter is necessary to avoid meaningless extreme values of K_a_/K_s_ estimates caused by low number of either synonymous or non-synonymous changes.

Logarithm of mean expression rate across 26 tissues [39] was used as a measure of expression rate in each paralog; coefficient of variation across tissues was used as a measure of non-uniformity of gene expression. An alternative measure of expression ubiquity is the expression evenness [41]; all conclusions reported below for expression CV hold (with the opposite sign) for the expression evenness, and only the results for the CV are reported. The central measure of log-mean expression and of expression CV between two paralogs was calculated as a simple mean; signed difference in mean and CV of expression and expression evenness is calculated as

$$d\left(E\right)=\left\{\begin{array}{c} E_{1}-E_{2},\mathrm{if}K_{a1}>K_{a2}\\ E_{2}-E_{1},\mathrm{if}K_{a2}>K_{a1} \end{array}\right),$$

where E_i_ is the expression mean or evenness of the i-th member of a paralogs pair and K_ai_ is its K_a_ value. Signed difference between SNPs K_a_/K_s_ ratio was polarized by divergence K_a_ in the same manner.

### Calculation of rates and asymmetries of sequence evolution

While the analysis of asymmetries of evolution rates may be advantageous, the choice of a good measure of asymmetry is not obvious, given that different pairs of paralogs vary greatly in their evolutionary rates and/or degree of divergence. A useful measure of sequence evolution rates’ asymmetry suitable for the comparison of numerous pairs of paralogs should possess the following properties: it should be equal to 1 if both clades accumulate changes at the same rate (values significantly below 1 implying unusually symmetric rates), the null-expectation should be rate-independent (i.e., should not depend on the total number or changes or portion of sites with changes), and it should be target size independent (i.e., should not be biased by different number of evolving sites in the two sequences being compared). It is also convenient to use a measure of asymmetry directly related to a commonly used statistic so that a statistical test of its deviation from the null expectation can be easily conducted. A commonly used measure of rates asymmetry is

$$A=\frac{{\left(N_{1}-N_{2}\right)}^{2}}{N_{1}+N_{2}},$$

(e.g., [42]), where N_1_ and N_2_ are the number of substitutions in clades 1 and 2. This measure does not satisfy these requirements because its null expectation decreases with the frequency of substitutions, and it does not account for possible differences in the sequence length in the two clades. Instead, we propose to use the square of normal deviate Z,

$$Z^{2}=\frac{{\left(p_{1}-p_{2}\right)}^{2}}{p\left(1-p\right)\left(\frac{1}{L_{1}}+\frac{1}{L_{2}}\right)},$$

where p_1_ and p_2_ are the frequencies of sites with substitutions in the two sequences of length L_1_ and L_2_ and

$$p=\frac{N_{1}+N_{2}}{L_{1}+L_{2}}$$

([43], Section 24.11). Z^2^ is numerically equivalent to Yates-corrected χ2 test ([43], Section 23.3) with the numbers of sites with and without substitutions in each of the two lineages as the elements of the 2x2 heterogeneity table.

Because we are calculating the substitutions by comparing each members of a pair of paralogs to their common ancestral state reconstruction, alignment lengths L_1_ and L_2_ do not have to be equal due to paralog-specific indels. When L_1_ = L_2_, Z^2^ = A/(1-p).

We investigated the behaviour of the quantity Z^2^ under various assumptions about the rates of evolution by simulating the evolution of homologous protein sequences when the null expectation of equal rates holds. We concluded (see Additional file 1: Figure S1 and Additional file 2: Figure S2) that, unlike that of the quantity A (data not reported), the null-expectation of Z^2^ is not biased by the degree of divergence between the sequences. On the other hand, unequal rates of evolution among sites brings the null expectation of Z^2^ below 1, while epistatic interactions among substitutions bias the null-expectation substantially above 1. Thus, in addition to possible asymmetry in substitution rates, any observed Z^2^ > 1 may reflect strong epistatic interactions; even if the two sequences are completely identical in terms of intrinsic selective constraint, if earlier substitutions make further substitutions more permissible, the rates will appear more different than one might expect under the assumption of rate equality. Thus, any significant Z^2^ >1 reported below can reflect two different causes difficult to discern: either stronger stabilising selection in one paralog than in the other, or strong epistatic selection operating in both paralogs.

For the purpose of regression analysis of the relationships between Z^2^ and expression and polymorphism data, the values of Z^2^ were log-transformed for the sake of normality; this necessitates removal of datapoints with of Z^2^ = 0, i.e., pairs of paralogs with exactly equal number of substitutions in each.

### Measures of radicality of amino acid substitutions

To evaluate how radical are amino acid substitutions observed in each clade, we calculated two measures of radicality: weighted average absolute difference in polarity |dPolarity| between the source and the destination amino acids and weighted average exchangeability, EX [44]. In both cases, posterior probabilities of a given substitution assigned to a given clade were used as weights. EX is a pairwise measure of amino acid exchangeability defined as a portion of missense mutations that do not result in a below-threshold reduction of protein function (stability of enzyme activity) estimated from the data on complete or selection-free random mutagenesis of 12 different proteins [44]. It strongly correlates with the absolute differences between polarity, hydrophobicity, charge, and side chain volume of the two amino acids and has been demonstrated to predict deleterious effects of single missense mutations better than a number of commonly used amino acid matrices. EX is a directional measure (i.e., EX_ij_≠EX_ji_), and it is therefore particularly useful in phylogenetic studies, in which substitutions observed between two sequences are polarised by the known ancestral state.

## Results

### Paralogs evolve faster and diverge more asymmetrically than their singleton orthologs

**Figure 2:**
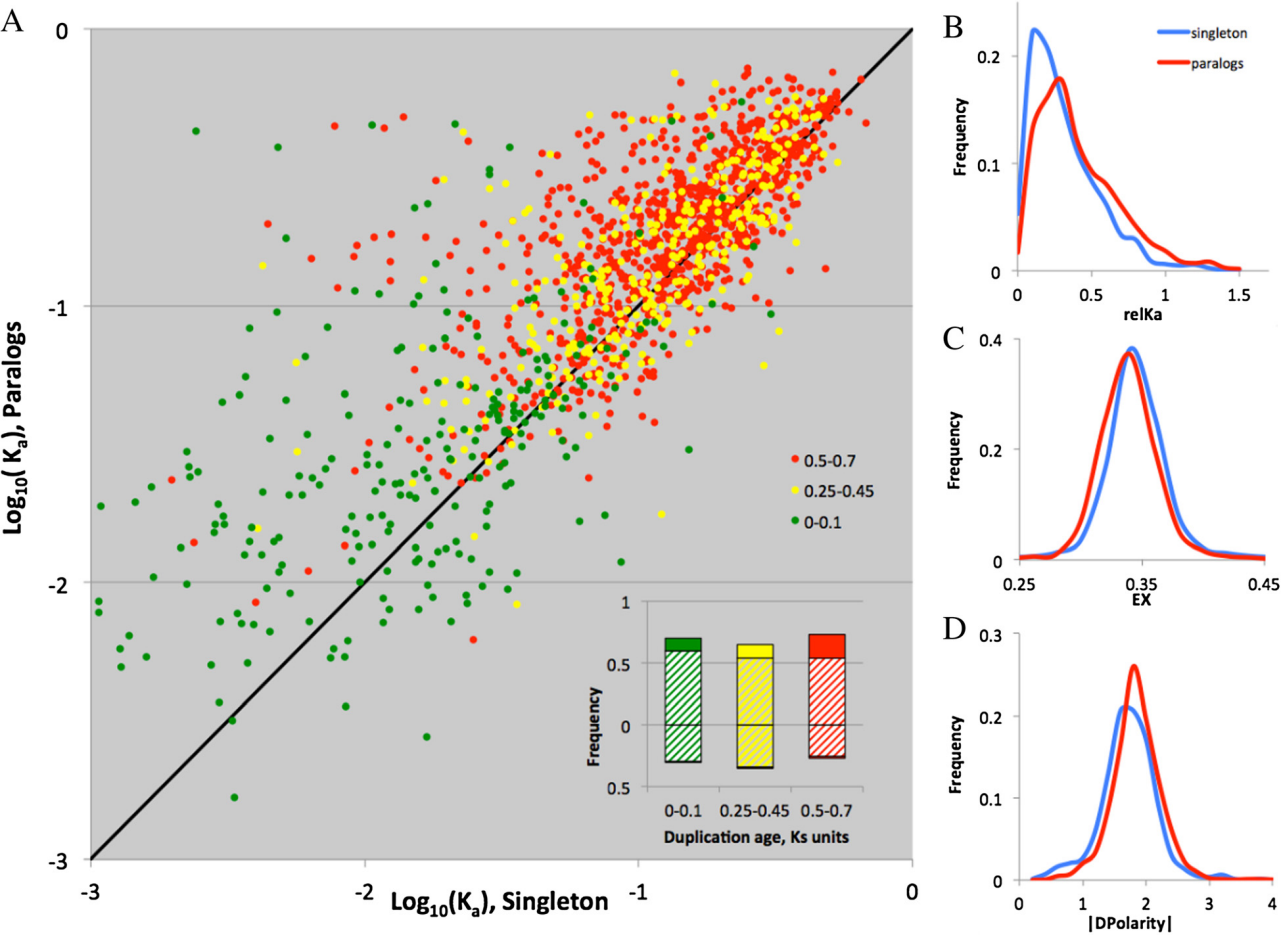
**Substitution rates in paralogs and their sister singletons. A**: Scatterplot of K_a_ (log scale) in paralogous vs. their nearest sister outgroup orthologous singleton clades. Colours of circles and lines indicate three classes of clade age measured in K_s_ units estimated for the speciation event. Inset: Frequency of triplets in which paralogs evolve faster (K_a, para_ > K_a, single_) than singletons (above 0 line) and vice versa (below 0 line). Filled portions of bars represent the frequency of triplets in which this difference is significant after Bonferroni adjustment (P_B_ < 0.05). **B-D**: Distribution of substitution rates (relK_a_), normalized by the upper bound estimate of duplication age **(B)**, absolute difference in polarity (|dPolarity|, **C)** and exchangeability **(D)** in paralogous (red) and singleton (blue) clades.

Duplicated genes demonstrated higher rates of amino acid substitutions than their singleton outgroup (Figure 2A, B, Additional file 3: Table S1). This result holds for all three duplication age groups defined by the age (in K_s_ units) of the preceding speciation serving as the upper-bound estimate of duplication’s age. This is a conservative test: because the speciation event defining the outgroup preceded the duplication event, for an unknown time, the paralogs in this analysis existed and accumulated substitutions as singletons. Moreover, paralogs evolved through more radical amino acid substitutions than their counterpart singletons, both for the radicality expressed as the absolute difference in polarity (|dPolarity|, Figure 2C) or as Exchangeability [44] (Figure 2D).

Numerous duplications demonstrated a significant asymmetry in frequency of amino acid substitutions between paralogous clades. Out of 1726 duplications analysed, 287 had the Yates-corrected χ2 test for asymmetry significant after Bonferroni correction (P < 0.05); 451 duplications had asymmetry test surviving false discovery rate correction [45] with q < 0.1. On average, paralogous clades diverged greater than did their singleton orthologous from the average of the two paralogs (Additional file 3: Table S1). This difference was more pronounced for older duplications and only marginally significant for the youngest (Ks < 0.051) ones. In contrast, the asymmetry of radicality was greater between orthologous than between paralogous clades and significant only in the youngest pairs of clades. Thus, paralogs diverge unexpectedly asymmetrically with respect to the numer of substitutions, but not with respect to radicality of these substitutions.

**Figure 3:**
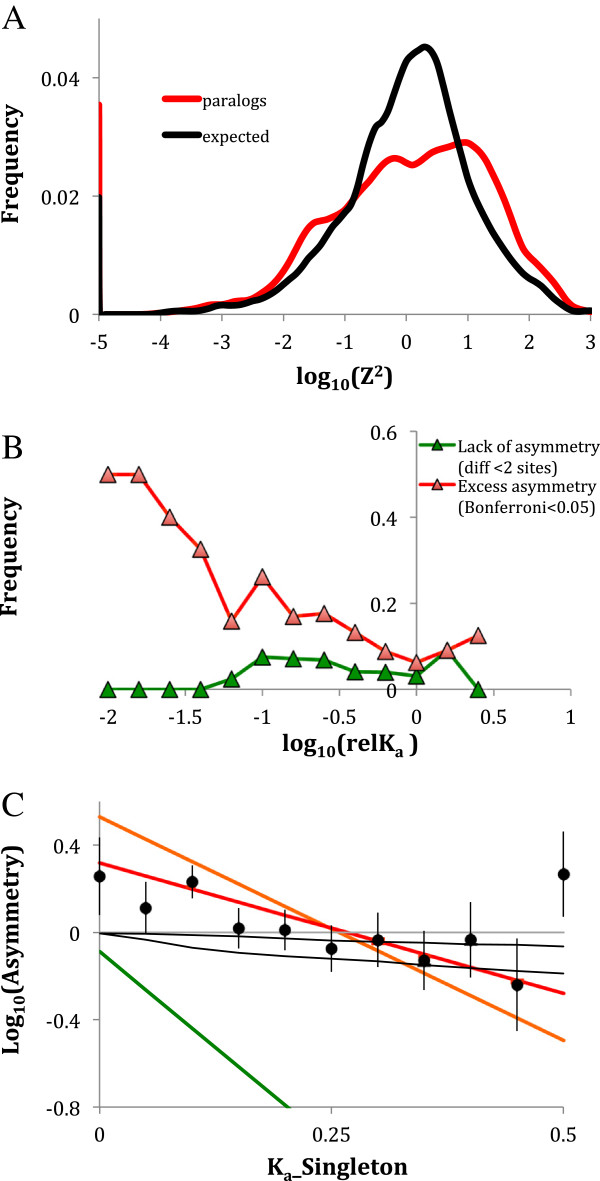
**Asymmetry of substitution rates in paralogs. A**: The distribution of K_a_ asymmetry in paralogous clade (red) relative to simulated asymmetry based on random placement of substitutions on the two clades (black). **B**: The portion of pairs of paralogs in which the observed asymmetry is either low (green) or significantly high (red, adjusted χ2 test P value <0.05 after Bonferroni correction). **C**. Substitution rates asymmetry of in paralogous clades vs. K_a_ values observed in the orthologous singleton clade, binned at 0.05 unit intervals on the logarithmic scale. Vertical bars are standard errors. Black lines: expected asymmetry in case of the shape parameter of gamma-distribution of substitution rate among amino acid sites 2 (top line) and 0.5 (bottom line). Linear regressions drawn through all points with different upper-bound duplication age estimate of duplication ages (colours as on Figure 2).

However, the comparison of mean asymmetry in paralogous and orthologous clades is misleading: the difference in the means notwithstanding, the distribution of asymmetries for paralogous clades is overdispersed relative to that for orthologous clades (contingency table test: P < 0.00001, Figure 3A). A large class of unusually symmetric pairs of paralogs is to be expected: firstly, some paralogs may have duplicated a significant and unknown time since the nearest preceding speciation and thus evolved for the unknown time as a single copy, not accumulating any differences; and secondly, even after duplication, paralogs may be evolving unusually symmetrically due to concerted evolution. These two effects are impossible to untangle in our design. Furthermore, the statisitical power of testing the hypothesis that the two paralogs are unusually symmetric (Z^2^ too small) is very low, as Z^2^ = 0 (equal number of substitutions in each paralog) is difficult to distinguish from the expected value Z^2^ = 1. None of the “unusually symmetric” pairs of paralogs survive Bonferroni correction for multiple tests of whether the observed Z^2^ is significantly below 1, except for those pairs in which there are equal numbers of substitutions in the two paralogs, resulting in a nominal P value of 0. Furthermore, unusually symmetric clades may be an artefact of the maximum likelihood failure to assign substitutions to specific branches. To illustrate this, Figure 3B shows the frequency of pairs of paralogs that differ by less than 2 substituted sites, as a function of relK_a_ of the singleton ortholog, without making a statement about statistical significance of such cases.

On the other hand, excessive asymmetry is observed in pairs of paralogs despite the two biases discussed above (Figure 3A, B). Excessive asymmetry was observed in gene families under the strongest stabilising selection, i.e., lower relK_a_ in the singleton ortholog (Figure 3B) and has a minimum at logarithm of relK_a_ = 0, i.e. in the pairs of paralogs in which the singleton orthologs evolves close to neutrality. Only about 5% of such duplications are significantly asymmetric after Bonferroni adjustment, while about 25% of duplications with singleton relK_a_ near 0.1 (−1 on the logarithm scale) are significantly asymmetric. The decrease of asymmetry with K_a_ can be caused by heterogeneity of evolution rates at different sites (see Methods and Additional file 1: Figure S1). Indeed, the observed mean asymmetry in classes of gene families with singleton K_a_ > 0.1 is consistent with reasonable assumptions about such heterogeneity (Figure 3C). Yet, the asymmetry in gene families with singleton K_a_ < 0.1 is significantly higher than the expected, indicating either relaxed selection in one of the paralogs or a strong epistatic interaction between substitutions (see below). As Figure 2B and age-specific regression lines on Figure 3C indicate, this observation is not caused by the effect of very young duplications but rather by the lower rate of substitutions in these gene families.

### Paralogs’ sequence evolution asymmetry correlates with gene expression and polymorphism asymmetry

**Figure 4:**
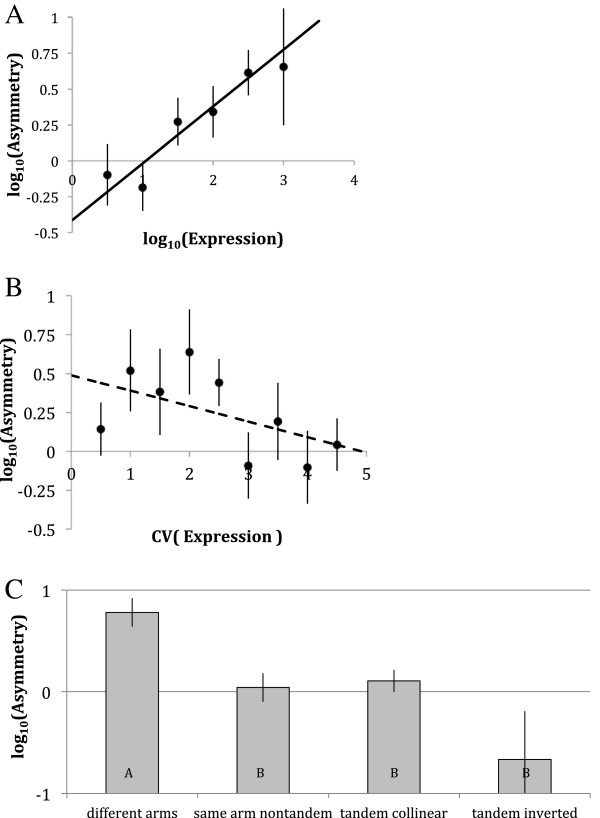
**The effects of expression level (A), expression evenness (B) and chromosomal location (C) on K**_**a **_**asymmetry of paralogous pairs in *D. melanogaster, *for which expression data are available **[34]**.** Vertical bars are standard errors. Regression lines are drawn based on all data points. **A**: regression coefficient = 0.397, P < 0.0004. **B**: regression coefficient = −0.0995, P < 0.11. **C**: mean log-transformed K_a_ asymmetry in paralogs located on different chromosomal arms, same arms but over 5 kb apart, less than 5 kb apart in collinear orientation and less than 5 kb apart in inverted orientation. Categories labelled with different letters are significantly different by Tukey test (P < 0.05).

If paralogs’ evolution asymmetry correlates with the selective constraint operating on the orthologous singleton genes, one would also expect a correlation between paralogs’ asymmetry and mean gene expression in these paralogs and with ubiquity of their expression, as these parameters are strongly correlated with selective constraint [46,47]. To test for this prediction we analysed asymmetry of substitutions in 175 duplications present in *D. melanogaster* genome. Among these, 38 duplications demonstrated a significant asymmetry of K_a_ rates between paralogs with false discovery rate q < 0.1 [45] (Additional file 3: Table S1). As predicted, there was a strong positive correlation between asymmetry of paralogs’ evolution and their mean expression rate (Figure 4A, Table 2) and a negative (Figure 4B, Table 2) correlation (with a statistically intangible hint of a maximum) with the CV of expression across 26 tissues sampled by FlyAtlas [39]. The average asymmetry of the lowest-expressed and the most non-uniformly expressed paralogs was not different from the random expectation. Thus, there was an emerging syndrome of asymmetrically evolving paralogs: these are orthologs of selectively constrained singletons that are, on average, highly and ubiquitously expressed. This observation is only true for the asymmetry of substitution rates; no significant relationship has been observed for duplications of any age between expression level parameters and radicality measures (data not reported).

**Table 2:** ANOVA of the effects of mean expression, CV of expression (continuous covariables) and chromosomal location (categorical effect) on substitution rate asymmetry between paralogs

**Source**	**DF**	**MS**	**F**	**P**
Log (Mean expression)	1	25.67	17.11	0.00005
CV (Expression)	1	13.69	9.13	0.00277
Location	3	8.55	5.7	0.0009
Error	264	1.5		

Both interactions are not significant and have been pooled.

One additional effect on paralogs’ evolution asymmetry has been identified: relative chromosomal location of the two paralogs (Figure 4C, Table 2). Paralogs located on different chromosomal arms are the only type of paralogs with the average asymmetry significantly above the null expectation. Inverted tandem duplications are the most symmetric, although not different from collinear tandem and same arm but distant duplications in Tukey test. Although the effects of mean expression, CV of expression and chromosomal location explained only 11% (adjusted R^2^) of the variance of asymmetry among pairs of paralogs, all three effects are highly significant (Table 2).

**Figure 5:**
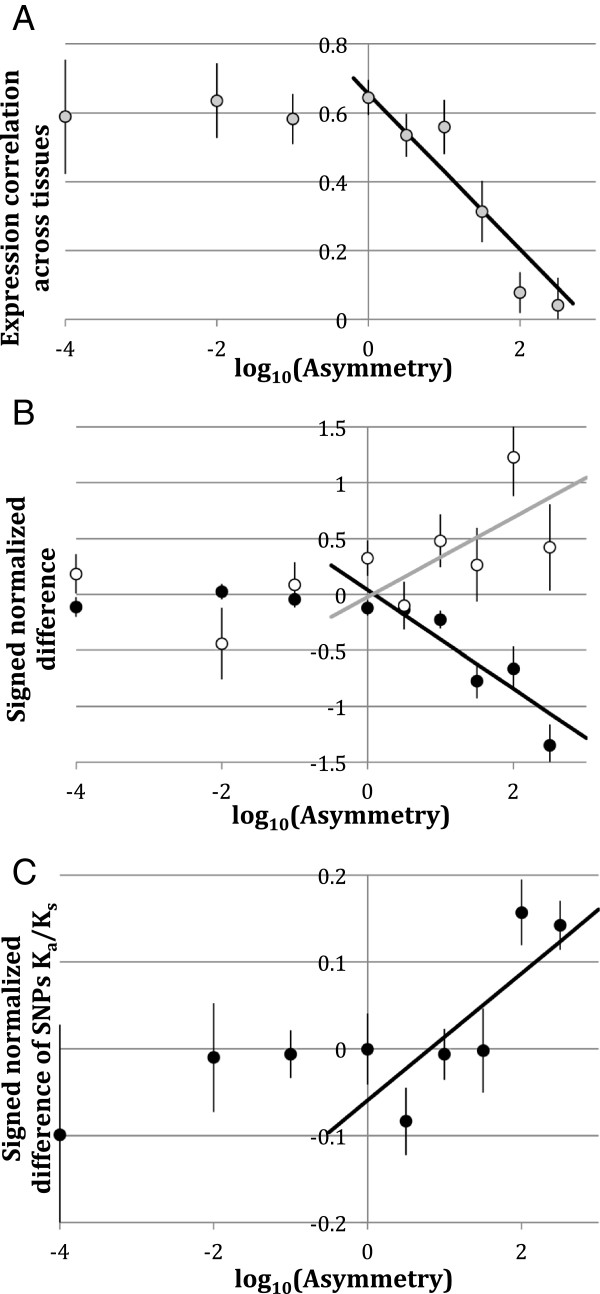
**Coefficient of correlation over 26 larval and adult tissues (A) and signed difference of mean gene expression (B, black circles), expression CV (B, open circles) and SNPs K**_**a**_**/K**_**s **_**(C) ratio in pairs of paralogs for which *D. melanogaster *expression data are available**[39]**, plotted against logarithm of K**_**a **_**asymmetry in the same pair (binned by 0.5 of a log**_**10 **_**unit).** Asymmetry is directionalized so that negative values correspond to the faster evolving paralog having lower mean expression level or lower evenness of expression. Vertical bars are standard errors. Regression coefficients (R) and significance levels for regression lines (linear regression on K_a_ asymmetry, both dependent and independent variables log-transformed, fitted for data points in which Z^2^ >1) are: **A** – R = −0.226; P < 1.20E-06; **B** - mean gene expression asymmetry: R = −0.442; P < 1.4E-08, gene expression evenness asymmetry: R = 0.356; P < 0.03; P < 0.004; **C** – R = 0.073; P < 0.002.

Do asymmetrically evolving paralogs diverge in their expression tissue specificity greater than more symmetrically evolving ones? Figure 5A demonstrates that while retained paralogs with low sequence evolution asymmetry (log Z^2^ < 0) retain a high (~0.6) coefficient of correlation across 26 tissues sampled from two developmental stages [39], those that diverged asymmetrically quickly lost such correlation, indicating that one of the members of the asymmetric pairs was also volatile in terms of gene expression specificity.

The question then arises whether it is the faster evolving member of such asymmetrically evolving pairs that is also the one that loses more expression average or uniformity. To test for this, we analysed signed asymmetry of log-transformed expression average and CV polarised by the sign of the asymmetry of the substitution rates. We observed that signed difference in mean expression between two paralogs (polarized by their K_a_) remained near 0 for symmetric pairs of paralogs and significantly decreased as the asymmetry increased (Figure 5B). The same figure demonstrates that the signed difference in expression CV increased with the asymmetry of substitution rates. Thus, in asymmetrically evolving pairs of paralogs the faster evolving member tended to lose both overall expression rate and the uniformity of expression across tissues, as indicated by the increase in expression rate CV. The same effect, graphically represented by the regressions on Figure 5B, can be confirmed by alternative statistical tests. Signed difference between log-transformed mean expression rates (faster paralogs – slower paralogs) was significantly less than 0 (Two-tailed t-test: df = 275, t = 6.53, P < 2E-10 for all pairs; df = 162, t = 7.39, P < 4E-12 for pairs with Z^2^ > 1 and df = 49, t = 6.60, P < 2E-8 for pairs with asymmetry surviving χ2 test with false discovery rate adjustment [39] at q < 0.1). Fisher’s exact test of the 2x2 contingency table (faster/slower evolution vs. higher/lower expression) resulted in P values of 0.0024 and 0.0007, respectively for all pairs and pairs with Z^2^ > 1. Two-tailed sign tests resulted in P-values of 0.001 and 0.0001, respectively. Likewise, the signed difference between expression CV (faster paralog – slower paralogs) was significantly greater than 0 (df = 275, t = 2.67, P < 0.0041; df = 162, t = 3.06, P < 0.0013 and df = 49; t = 2.58, P < 0.006 for all pairs, pairs with Z^2^ > 1 and pairs surviving false discovery rate adjustment for asymmetry test, respectively). However, non-parametric tests (FET and sign) were not significant for the signed difference of expression CV. The significant results reported here remained robustly significant when false discovery rate correction with q < 0.1 was used as a criterion of high asymmetry instead of the formal cut-off of Z^2^ > 1 (data not shown). (Note: the fact that the number of degrees of freedom in the tests for paralogs pairs with Z^2^ > 1 and the number of duplications analysed (Table 1) are the same (162) is a coincidence). Pairs of paralogs with false discovery rate adjustment q < 0.1, along with their expression and polymorphism data, are listed in Additional file 4: Table S2.

Does the faster evolving member of a pair of paralogs also accumulate higher frequencies of polymorphisms? Figure 5C demonstrates that, in the pattern already presented on Figure B, the average difference between SNPs K_a_/K_s_ estimates (faster paralogs – slower paralogs) is, predictably, close to 0 for symmetrically evolving paralogs pairs but significantly increases in the paralog pairs with the highest asymmetry. Here, the t-test of signed difference between K_a_/K_s_ estimates is significant only for the pairs with rates’ asymmetry surviving false discovery rate adjustment with q < 0.1 (df = 45, t = 5.11, P < 4E-6).

## Discussion

We analysed rates and asymmetries of substitution rates in duplicated genes in the 12 *Drosophila* genomes using the nearest non-duplicated ortholog as the outgroup and reference. We demonstrated that paralogs tend to evolve faster than their singleton orthologs (Figure 2), that this effect is largely independent of the duplication age, and that not only are there more substitutions per site occurring in duplicated genes than in singletons but that these substitutions are also more radical (Figure 2C, D). Although this observation has been made many times in the past [6,9,22,23,26], this is, to the best of our knowledge, the first such demonstration with the use of the nearest orthologous singleton as a reference and the first such demonstration on the material of 12 closely related genomes. We further demonstrate that the distribution of substitution rates asymmetry between paralogs is significantly overdispersed, with both unusually symmetric pairs and unusually asymmetric pairs observed more frequently among paralogous clades than among orthologous clades (Figure 3). The unusually symmetric pairs of paralogs are difficult to individually validate statistically and can be readily explained as being an artefact of the unknown period of evolving as a single gene between the speciation and duplication events, or an artefact of the failure of our maximum likelihood procedure to reliably assign substitutions to paralogous clades, or as a result of concerted evolution. Unusually asymmetric pairs of paralogs, on the other hand, are easy to subject to a statistical test of such asymmetry significance and are interesting because high asymmetry of substitution rates may be an indication of relaxed stabilising selection or accelerated positive selection in one but not the other copy.

However, two genes can evolve asymmetrically for a fundamentally different reason: epistatic interactions among substitutions at different amino acid sites, an effect largely overlooked by previous interpretations of sequence evolution asymmetry. If a particular substitution makes further substitutions at other sites, previously prevented by stabilising selection, more likely, the binomial model of distribution of substitution events into the two clades does not apply, and the null expectation of the observed asymmetry can be substantially greater than 1 even though selective constraint in the two copies is exactly identical (Additional file 2: Figure S2). Thus, all results of this study and previous studies that concern rates’ asymmetry should be interpreted as simply detection of higher rate of evolution in one paralog copy than in the other without an implication that such higher rate is caused by innate asymmetry in selection pressures and not by random relaxation of stabilising selection in the copy that happened to incur an epistatically acting substitution. Yet, with this in mind, all the conclusions we arrive at in the further discussion remain valid. Similarly, the apparent lack of asymmetry relative to the binomial expectation can be readily explained by unequal substitution rates per site (Figure 3C and Additional file 1: Figure S1). Thus, any asymmetry between sequence evolution rated between two genes has to be strong enough to be detectable despite the effect of unequal substitution rates.

Average asymmetries we observed were close to the null expectations, but numerous individual pairs of paralogs demonstrated a significant asymmetries. We were able to observe at the following patterns in the pairs of paralogs that are likely to evolve asymmetrically. Firstly, such pairs tend to have orthologs showing lower overall substitution rates (Figure 3C). This effect is not caused by lower evolutionary age of the duplication in question but rather by stronger selective constraint (Figure 3B, C). Gene families under stronger stabilising selection tend to generate more asymmetric duplications despite the prediction that highly conserved genes are more likely to be maintained in duplicated state by gene conservation [5-7]. This effect is more pronounced in older duplications than in the youngest ones (Figure 3C). In fact, the regression line on Figure 3C for the youngest substitutions has the intercept not significantly different from 0, indicating that even in young duplications in the most constrained gene families the rate asymmetry is low. This observation is in a general agreement with stabilizing selection acting to maintain similar sequences and possibly functions in highly constrained paralogs. Furthermore, the fact that the asymmetry of paralogs’ evolution is less pronounced in younger duplications is consistent with the idea that the divergence of paralogs with high sequence similarity is constrained by concerted evolution, while pairs of paralogs that have diverged sufficiently to escape the homogenizing effect of gene conversion or unequal crossing over continue to accumulate substitutions to become even more divergent [48].

Secondly, on the material for *D. melanogaster*, for which detailed gene expression data are available, we demonstrated that the most asymmetric duplications tend to be observed in gene families with the highest mean expression (Figure 4A) and with the lowest CV of expression across 26 tissues sampled in two developmental stages [39], i.e., with the highest ubiquity of expression (Figure 4B). This is in contradiction to a previously observed result in yeast [49], where the most symmetric pairs (i.e. pairs most likely to be maintained by gene conservation) tend to have the highest expression rate. Moreover, these observations suggest that widespread neofunctionalization may be occurring more frequently in household genes with high and ubiquitous expression. However, they can also be explained by unfinished or incomplete pseudogenization in one of the duplicated copies of such genes. Indeed, further analysis demonstrated patterns of expression and polymorphisms consistent with unfinished pseudogenization (see below).

Finally, the most asymmetric pairs of paralogs tended to be found among non-tandem duplications (Figure 4C). This observation is consistent with two explanations: firstly, neighbouring duplicates are more likely to experience concerted evolution due to gene conversion or unequal crossing over, and secondly, distant duplications are more likely to occur by retrotransposition, resulting in one of the paralogs being deprived of its promotors or other cis-regulatory chromosomal context, predestining this copy for pseudogenization. In addition, inverted tandem duplications are to be expected to be the most symmetric, as crossing over in such repeats results in gene conversion with retention of both copies. Meanwhile, crossing over between collinear repeats results in gene conversion accompanied by a loss of one of the copies. Tandem duplications also were showing less expression divergence than non-tandem ones (data not shown), corroborating previous findings in plants [50,51], rodents [51,52] and *Drosophila*[10,51,53].

In a pattern corroborating the previous findings in yeast [25] and rodents [9] higher sequence divergence between paralogs correlates with higher divergence of tissue-specific expression (Figure 5A). The faster evolving member of the pair was likely to show lower mean expression and higher coefficient of variance of expression across tissues, making it “less household” than the slower evolving copy (Figure 5B). This is consistent with the recent finding [10] that the faster evolving paralog (“child” in the terminology of [10]) attains a significantly higher tissue specificity. Yet, as we argue here, this pattern is not necessarily indicative of neofunctionalization [10], as it is also fully consistent with gradual decay of expression during pseudogenization.

The faster evolving member of the most asymmetric pairs also had, on average, a significantly higher K_a_/K_s_ ratio for segregating SNPs than its slower evolving counterpart, indicating either relaxed stabilising selection or positive selection acting on these loci (with caveats, [54]). While some of these observations are consistent with other modes of duplication retention, we believe that unfinished pseudogenization is likely to be the most parsimonious explanation for the observed patterns in a large number of *D. melanogaster* paralog pairs. This indicates that numerous duplicated copies “on their way out” may still be recognized as functional genes by the bioinformatics approaches [35] and may still be expressed according to FlyAtlas data [39]. Only one of the significantly asymmetric pairs of *D. melanogaster* paralogs (Additional file 3: Table S1) has a member (FBgn0036646, CR18217) that is identified as a pseudogene in the current FlyBase annotation (note that it does have expression and polymorphism data). It has been well established that pseudogenes, including those resulting from gene duplication can persist in the genome for a long time, retaining transciption [55]. While no comprehensive theory of time until pseudogenization exists, one may expect that selection in favour of complete loss of expression or complete loss of a pseudogene altogether is weak and degenerative changes are accumulating by drift, thus allowing slow and gradual decay. It is worth noting that the paralog with faster coding region evolution also typically being the one losing more expression is consistent with the theoretical finding [56] that less ubiquitously expressed genes are likely to show a higher rate of evolution due to the accumulation of deleterious mutations. In that study, environmentally specialized genes were considered, but it is reasonable to assume that tissue specialization may also result in lower selective constraint.

Is the fate of the copy with a faster rate of substitutions, lower expression, and more frequent non-synonymous polymorphisms sealed? Perhaps not, if it is allowed to linger in the genome for a long time despite partial loss of expression and accumulation of harmful mutations. Such lingering semi-pseudogenes may be an important resource for eventual neofunctionalization or subfunctionalization. However, because neofunctionalization may also result in sequence evolution asymmetry while subfunctionalization may also result in loss of overall expression or expression uniformity in one of the copies, it is difficult to detect these processes by the genome-wide analysis of sequence and expression divergence as any signal from these processes occurring in a particular gene is going to be indiscernible from similar patterns caused by unfinished pseudogenization alone. A clear-cut demonstration of the signature of neo- or subfunctionalization would therefore require a detailed gene-specific structural and expression analysis [17-19,57].

## Conclusions

We observed higher rates and radicality of amino acid substitutions in duplicated clades of *Drosophila* genes than in their nearest singleton orthologous outgroup, which indicates relaxed selective constraint in duplicated genes. The substitutions were often distributed asymmetrically in the two paralogous clades: paralogs diverged faster than their nearest ortholog from either of them. Such paralog asymmetry was higher in gene families with higher selective constraint, higher gene expression and higher expression ubiquity. The faster evolving paralog in each pair had, on average, lower and less ubiquitous expression and higher frequency of non-synonymous polymorphisms than its slower counterpart. All these observations are consistent with the hypothesis that many duplications in *Drosophila* are retained despite relaxation of stabilizing selection in one of the copies, i.e., due to unfinished or incomplete pseudogenization.

## Reviewers’ comments

### Reviewer 1: Jia Zeng, School of Biology, Georgia Institute of Technology (nominated by I. King Jordan)

The authors present an analysis of asymmetry in sequence evolution, expression level, expression specificity and segregating non-synonymous polymorphisms in paralogs present in 12 *Drosophila* genomes by using singleton orthologs as controls. Based upon these analyses, the authors conclude that paralogs evolve faster and diverge more asymmetrically than their singleton orthologs, and the sequence evolution asymmetry correlates with gene expression and polymorphism asymmetry. While there are some interesting results in this manuscript, the questions listed below need to be well addressed by the authors.

### Major comments

My primary critique is that the purpose and biological novelty of this manuscript are unclear. First, the major finding in this work is that paralogs tend to evolve faster than their singleton orthologs. But this result has been found many times in previous studies (see authors’ reference [6,18,19], as well as Kim and Yi 2006 MBE 23: 1068 and references therein).

Second, as it is pointed out in the manuscript, the analysis of sequence evolution asymmetry is prone to false negatives by comparison of aligned amino acid sequences. However, even though this study incorporate the analysis of the rate and asymmetry in gene expression and polymorphisms, this study didn’t improve the assessment of asymmetry in sequence evolution since the methodology used in this study is still based upon the sequence alignment.

Authors’ response: *Certainly there have been several papers recently published with similar approach and similar results; however, few analyse sequence divergence in parallel with expression divergence (e.g.,*[9,10,25]*) and none, to our knowledge, incorporate polymorphism data. To address this criticism we cited several works either regretfully overlooked in or published since the previous version. We also changed the title of the paper to emphasize our most important findings.*

Third, the main metric the authors to use quantify sequence evolution asymmetry needs to be better described. According to the description in the “Methods” section, this measure is equal to 1 if both clades accumulate changes at the same rate. However, in the most simple case when there are equal numbers of substitutions in both branches, Z2 will be 0 (because p1 = p2). Even though the authors discuss that the null expectation of Z2 would be affected by the equalities of the evolution rates among sites, in my opinion these points were not clearly illustrated, and certainly failed to persuade this reviewer. Also some of these discussions should be moved to the main text rather than supplementary material.

Authors’ response: *It is certainly our fault that we did not make it clear enough in the previous version of the manuscript, but the reviewer is incorrect in his assessment of the expectation of our asymmetry measure, Z*^*2*^*. His assertion that the expected value is 0 would have been correct if we used signed asymmetry. But in the case of an unsigned measure the expectation is > 0, and exactly 1 in the case of Z*^*2*^*. Equal numbers of substitutions in both branches, indeed the case with the highest individual expectation, is not, however, the only possible outcome because of random variance in the number of substitutions per branch. Z*^*2*^*captures this variance.*

### Minor comments

Abstract, “Background” section: The first sentence in the “Background” section need editing for grammar. There are too many words of “when”.

Abstract, “Background” section: Delete the word of “in” in this sentence: “b) in genes with high expression level”.

Methods, “Merging with expression and polymorphism data” Paragraph 1: Delete the word of “new” in “Table 1 new”.

Methods, “Calculation of rates and asymmetries of sequence evolution”: This sentence “Z2 is numerically equivalent to Yates-corrected ×2 test with no fixed margins with the numbers of sites with and without substitutions in each of the two lineages as the elements of the 2×2 heterogeneity table.” needs revision.

Results, “Paralogs evolve faster and diverge more asymmetrically than their singleton orthologs” Paragraph 1: The authors claim “Paralogs evolve faster than their singleton orthologs” based upon the observation of larger Ka in paralogs. I wonder whether the authors perform similar analysis in Ks, which is the synonymous rate, and lead to the similar result.

Results, “Paralogs’ sequence evolution asymmetry correlates with gene expression and polymorphism asymmetry”: In the last paragraph, the authors describe the higher frequencies of polymorphisms in the faster evolving paralog. It seems that the authors use the SNPs Ka/Ks to estimate the frequencies of polymorphisms, but there is no description about how these values are calculated in the “Method” section. In addition, the authors need to consider that SNP Ka/Ks is notoriously inefficient or misleading to use (e.g., Kryazhimsky and Plotkin, PLoS Genetics 2008 4:e1000304).

Figure 2: The axis titles are missing for the inset figure of Figure 2A. Please revise.

Figure 3: In the Figure 3B, if the value in the y-axis represents the portion of pairs of paralogs, the summation for the “excess asymmetry” category is obviously exceed 1. In the Figure 3C, the colors here need revision. According to the figure legend, the linear regression lines here have the same color code in Figure 2 but the orange color doesn’t exist in Figure 2. Besides, black dots here need more clarification in the figure legend. At last, the authors should explain more about the black lines. i.e., what is the value of 2 (top line) and 0.5 (bottom line), and why these values are chosen?

Figure 4: In the figure legend, the authors should give more description about the label of “A” and “B” in the bars of Figure 4C.

Figure 5: This sentence in the figure legend: “ Asymmetry is directionalized so that negative values correspond to the faster evolving paralog having lower mean expression level or lower evenness of expression”, need revision and is not appropriate in the legend since it is more result-based description. In addition, in Figure 3B, the color legend should embedded with the figure to make it consistent with other figures.

Discussion, paragraph 4: Since the result of “ most asymmetric duplications tend to be observed in gene families with the highest mean expression” is inconsistent with the previous study, then why both observations are consistent with the prediction of “widespread neofunctionalization occurring more frequently in household genes with high and ubiquitous expression”?

Discussion, paragraph 6: A significantly higher Ka/Ks ratio for SNPs in faster evolving member of paralogs may not necessarily indicate a relaxed selection constrain. It could also due to positive selection in these loci.

### Reviewer 2: Fyodor Kondrashov, Centre for Genomic Regulation, Barcelona

This is straightforward but broad analysis that aims to make some of the usual observations on the evolutionary properties of duplicated genes. There are several advantages for this paper, which includes the breadth of data obtained for the analysis, a good selection of genes for the paralog-orthologue comparison and what I thought was a good statistical analysis of rate asymmetries.

A potential weakness, but not a flaw, of the paper is that it does not formally relate the observations to mathematical models of the expected time to pseudogenization. I understand, however, that the authors may feel that this is possibly beyond the present analysis.

Perhaps the most substantial weakness is the lack of a clear demonstration of the use of synonymous rates of evolution. It is mentioned that previously used estimates across the phylogeny are used. However, the difference between Ka/Ks and relKa are not apparent from a first read of the manuscript. Furthermore, I think a figure on which types of topologies for duplicated genes were included for the analysis would greatly simplify the current description. Examples of used and discarded topologies would be a useful figure (perhaps more useful than the present Figure 1).

Authors’ response: *We followed this suggestion.*

A similar analysis done in mammals before should be cited:

Pegueroles C, Laurie S, Albà MM. Accelerated evolution after gene duplication: a time-dependent process affecting just one copy. Mol Biol Evol. 2013 Aug;30(8):1830–42.

Minor errors to correct.

Page 2: “eliminate this confounding is to compare”

Table 1 new – in several places.

Yampolsky and Stoltzfus, 2005 - right before the Results section.

First paragraph of Discussion: “observation has been made many times in the past faster [6,18,19]”.

### Reviewer 3: Yuri Wolf, National Center for Biotechnology Information

Yampolsky and Bouzinier trace the evolution of paralogs derived from duplications that occurred within the 12-species tree in *Drosophila* genus. They analyze the evolution rate and its asymmetry and find correlations between the rates, expression profiles and polymorphism frequency. Their results show a prevalent pattern of mutation and expression asymmetry between the paralogs that they interpret as evidence for widespread unfinished pseudogenization (with caveats).

The work is generally well-designed and adequately described; the results are interesting (although, unfortunately, less conclusive than one could hope, as alternative explanation can’t be ruled out).

This reviewer has one concern regarding the experimental design that might require some re-analysis. The asymmetry of evolution along the paralogous genes is compared to the asymmetry of the evolution between the singleton ortholog and the (average) evolution along the clade that contain the duplication. The asymmetry between the orthologous genes is used as the control under the assumption that this asymmetry is generated by random variation of evolution rates. The asymmetry of evolution of paralogs is then compared to the asymmetry of evolution of orthologs to detect the possible signal of unequal selection acting upon the paralogs.

First, purely technically, if both paralogs are evolving under relaxed selection (as many post-duplication scenarios suggest), then one expect that the clade with paralogs to accumulate more mutations (even averaged between the paralogs) that the clade carrying the singleton ortholog. This would exaggerate the ortholog asymmetry and, potentially, mask the signal from the differential selection on paralogs.

More importantly, both paralogous lineages evolve in the same genome(s), while it’s singleton ortholog counterpart evolves in a different clade of organisms. Thus any genome-wide acceleration of deceleration of evolution (global deviation of molecular clock) in either of the clades would affect the perceived asymmetry of orthologous evolution. Such deviations exist (most famously, in murine-primate comparison in mammals) and also are a factor in the evolution of *Drosophila* species (PMID:17989260). Thus the observed distribution of asymmetry of orthologous evolution really depends on the clades that happened to be chosen for the comparison. Some combinations of clades show a very clock-like behavior while others are very asymmetric. Therefore this asymmetry shouldn’t be used as a control.

I would suggest using the simulated distribution of paralog asymmetries, created by randomly assigning the inferred mutations to the paralogous clades, as a neutral background (such simulation would preserve the number and the character of mutations in the context of the same gene lengths) while indicating the expected range of variation under the underlying symmetric evolutionary process.

Authors’ response: *the reviewer is of course correct that this comparison is not valid for more than one reason. It has been removed from the analysis. Instead, as suggested by the reviewer, the asymmetry between paralogs is compared to the simulated asymmetry based on random assignment of substitutions to paralogous clades.*

Minor concerns (mostly technical and stylistic):

General: An additional panel for Figure 1, schematically depicting the comparisons of rates involved in this work, would have helped many readers.

p. 5. “A wholesale analysis of rates”. Maybe “A gene-wide analysis of rates”?

p. 9. “excluded duplications present in the root ancestor”. It seems that the root in this context refers not to the root of the 12 species, but to the speciation node immediately preceding the duplication. Either way, this needs to be made clear.

p.9. “Table 1 new”. Probably a leftover from the manuscript editing.

p. 10. “Only paralogs in which there were at least three synonymous and at least three non-synonymous SNPs were retained for the polymorphism analysis”. The authors should convince the readers that this procedure won’t create a bias towards particularly highly-variable genes.

p. 11. “A commonly used measure of amino acid substitution rates asymmetry”. Citation needed.

p. 13. “This result holds for all three duplication age groups defined by the age (in Ka units)”. Probably a typo. Other occurrences in the text indicate that the age is measured in Ks units.

p. 18. “The unusually symmetric pairs of paralogs are difficult to individually validate statistically and can be readily explained by either the artefact of the unknown period of evolving as a single gene between the speciation and duplication events”. This is unclear. Since the asymmetry is calculated from comparison of two clades, the time during which they weren’t separate should affect only the absolute number of accumulated mutations, not their distribution between the clades? How this could make a pair of paralogs “unusually symmetric”?

Authors’ response: *Clearly it was the authors’ fault that this paragraph sounded confusing. This comment would have been valid if we were measuring the number of changes between extant sequences (in which case asymmetry would have been simply divergence). We, on the contrary, are measuring asymmetry by comparing the number of substitutions since common ancestor in each branch. Thus, prolonged period during which of two paralogs really were a single gene will indeed result in underestimated asymmetry. We clarified this in the text.*

p. 28, ref #48. Possibly incomplete reference or wrong format.

Figure 2A and 2B. The authors should check if plotting the data in log format makes a visually better picture (it is often so with evolutionary distances).

Figure 2B,C,D and 3A. Narrow bars make the pairs of distributions difficult to distinguish. The authors should explore the option to plot kernel-estimated probability densities instead of the binned frequencies.

Authors’ response: *We followed all suggestions regarding figures.*

## Abbreviations

CV: Coefficient of variation; DGRP: Drosophila genetic reference panel [34]; EX: Amino acid exchangeability [37]; relKa: Frequency of non-synonymous substitutions normalized by branch length; SNP: Single nucleotide polymorphism.

## Competing interests

The authors declare that they have no competing interests.

## Authors’ contributions

LY designed the study, analyzed the aggregate data and wrote the manuscript. MB developed software, analyzed the data and contributed to manuscript preparation. Both authors read and approved the final manuscript.

## Authors’ information

LY is an associate professor at East Tennessee State University. MB is a software developer at InterSystems Corp.

## Supplementary Material

Additional file 1: Figure S1 Results of simulated of evolution of a pair of homologous with equal substitution probabilities and gamma-distributed substitution rates per site. Asymmetry of substitution rate (Z2; shown on logarithm scale) was calculated after the members of the pair achieved the average substitution rate Ka. Shape parameter of gamma distribution varied from 20 (nearly equal rates) to 0.5 (strongly leptokurtic distribution). Black lines: both homologs genes are 500 codons long. Red lines: one of the homologs is 500 codons, the other 400 codons long. Null expectation of asymmetry is 0 (on logarithm scale). Non-uniformity of substitution rates results in the null expectation of asymmetry decreasing with Ka. Unequal gene length reverses this effect for leptokurtic distributions and has no effect in case of equal or nearly equal rates. Click here for file

Additional file 2: Figure S2 Results of simulated evolution of a pair of homologous with equal substitution probabilities and gamma-distributed substitution rates per site (k = 20 corresponding to equal substitution rates, solid lines; k = 0.5 corresponding to a strongly leptokurtic distribution, dotted lines) with various strength of epistatic effects between substitutions. Each gene can contain either one (purple, green) or 5 (red, blue) sites, which, if incurring a substitution, increase substitution rates at other sites by 10% (blue, green) or by 100% (red, purple). Mild epistasis does not strongly affect the null expectation of asymmetry until very high Ka values. However, even moderately strong epistasis can result in very high asymmetry values even with very small Ka. Click here for file

Additional file 3: Table S1 Matched pairs two-tailed t-test of the hypotheses of relaxed constraint in paralogs than in singletons (Top) and higher asymmetry of evolution of clades resulting from duplication events than outgroup clades resulting from speciation events (Bottom) among all clades and by age class of the outer speciation event, measured in Ks units estimated at the nearest speciation preceding the duplication event. Asymmetry measures are Z2 for Ka; absolute normalized difference for |dPolarity| and EX. Negative t-values in Ka and |dPolarity| and positive values for the EX mean faster/more radical evolution in paralogs than in orthologous singletons. The same signs for the asymmetry indicate that the divergence between two paralogous branches is greater than the average of the divergence between their orthologous singleton and each of them. 2-tail P values are reported. Click here for file

Additional file 4: Table S2 Pairs of D. melanogaster paralogs demonstrating a significant rate asymmetry (false discovery rate adjustment (Benjamini & Yekutieli, 2001) at q<0.1). Gene family IDs correspond to those on http://www.indiana.edu/~hahnlab/fly/DfamDB/drosophila_frb.html. Pairwise columns shown: r – coefficient of correlation of expression level over 26 tissues; dME – signed difference in log mean expression level polarized by the differences in Ka; dCVE – signed difference in coefficient of variation of expression level in 26 tissues polarized by the differences in Ka; dKa/Ks_poly – signed difference in SNPs Ka/Ks polarized by the differences in divergence Ka. Click here for file
