# Supplementary material for: Faster evolving Drosophila paralogs lose expression rate and ubiquity and accumulate more non-synonymous SNPs
Source: Biol Direct. 2014 Jan 17;9:2. doi: 10.1186/1745-6150-9-2 (PMC3906896; doi:10.1186/1745-6150-9-2)

Additional file 1: Figure S1. Results of simulated of evolution of a pair of homologous with equal substitution probabilities and gamma-distributed substitution rates per site. Asymmetry of substitution rate (Z^2^; shown on logarithm scale) was calculated after the members of the pair achieved the average substitution rate K_a_. Shape parameter of gamma distribution varied from 20 (nearly equal rates) to 0.5 (strongly leptokurtic distribution). Black lines: both homologs genes are 500 codons long. Red lines: one of the homologs is 500 codons, the other 400 codons long. Null expectation of asymmetry is 0 (on logarithm scale). Non-uniformity of substitution rates results in the null expectation of asymmetry decreasing with K_a_. Unequal gene length reverses this effect for leptokurtic distributions and has no effect in case of equal or nearly equal rates.


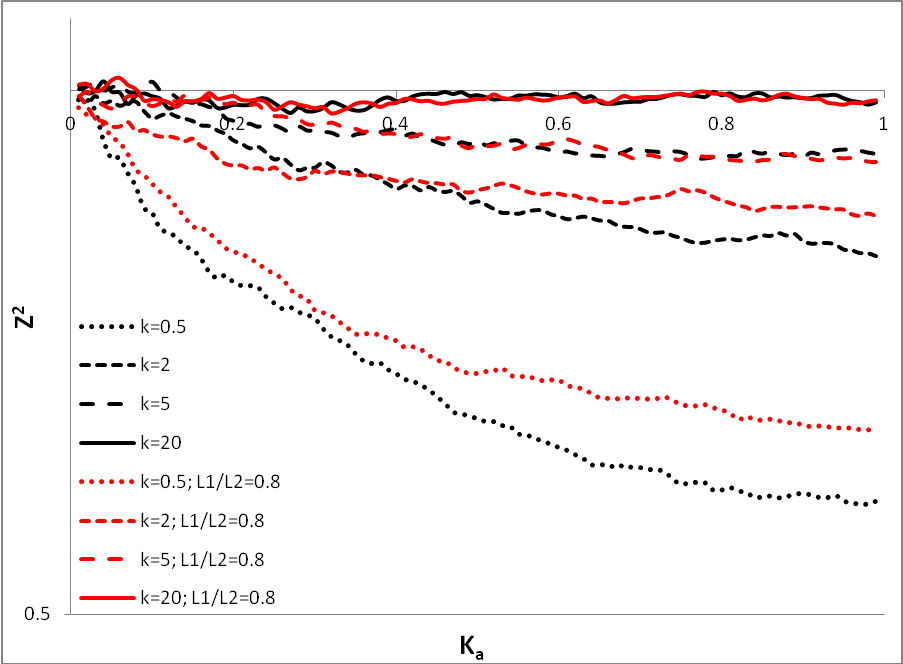

Supplement: Additional file 1: Figure S1 — Results of simulated of evolution of a pair of homologous with equal substitution probabilities and gamma-distributed substitution rates per site. Asymmetry of substitution rate (Z2; shown on logarithm scale) was calculated after the members of the pair achieved the average substitution rate Ka. Shape parameter of gamma distribution varied from 20 (nearly equal rates) to 0.5 (strongly leptokurtic distribution). Black lines: both homologs genes are 500 codons long. Red lines: one of the homologs is 500 codons, the other 400 codons long. Null expectation of asymmetry is 0 (on logarithm scale). Non-uniformity of substitution rates results in the null expectation of asymmetry decreasing with Ka. Unequal gene length reverses this effect for leptokurtic distributions and has no effect in case of equal or nearly equal rates. [file 1745-6150-9-2-S1.doc]
