# Supplementary material for: Faster evolving Drosophila paralogs lose expression rate and ubiquity and accumulate more non-synonymous SNPs
Source: Biol Direct. 2014 Jan 17;9:2. doi: 10.1186/1745-6150-9-2 (PMC3906896; doi:10.1186/1745-6150-9-2)

Additional file 2: Figure S2. Results of simulated evolution of a pair of homologous with equal substitution probabilities and gamma-distributed substitution rates per site (k = 20 corresponding to equal substitution rates, solid lines; k = 0.5 corresponding to a strongly leptokurtic distribution, dotted lines) with various strength of epistatic effects between substitutions. Each gene can contain either one (purple, green) or 5 (red, blue) sites, which, if incurring a substitution, increase substitution rates at other sites by 10% (blue, green) or by 100% (red, purple). Mild epistasis does not strongly affect the null expectation of asymmetry until very high Ka values. However, even moderately strong epistasis can result in very high asymmetry values even with very small K_a_.


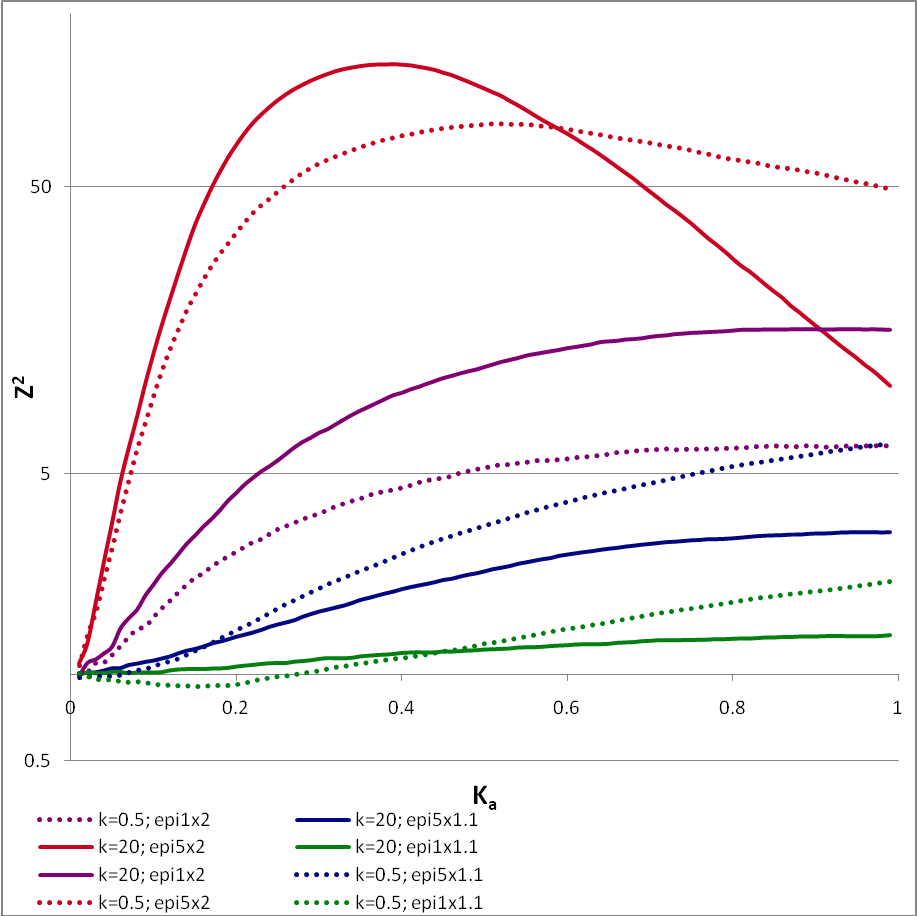

Supplement: Additional file 2: Figure S2 — Results of simulated evolution of a pair of homologous with equal substitution probabilities and gamma-distributed substitution rates per site (k = 20 corresponding to equal substitution rates, solid lines; k = 0.5 corresponding to a strongly leptokurtic distribution, dotted lines) with various strength of epistatic effects between substitutions. Each gene can contain either one (purple, green) or 5 (red, blue) sites, which, if incurring a substitution, increase substitution rates at other sites by 10% (blue, green) or by 100% (red, purple). Mild epistasis does not strongly affect the null expectation of asymmetry until very high Ka values. However, even moderately strong epistasis can result in very high asymmetry values even with very small Ka. [file 1745-6150-9-2-S2.doc]
