# Supplementary material for: Faster evolving Drosophila paralogs lose expression rate and ubiquity and accumulate more non-synonymous SNPs
Source: Biol Direct. 2014 Jan 17;9:2. doi: 10.1186/1745-6150-9-2 (PMC3906896; doi:10.1186/1745-6150-9-2)
Supplement: Additional file 3: Table S1 — Matched pairs two-tailed t-test of the hypotheses of relaxed constraint in paralogs than in singletons (Top) and higher asymmetry of evolution of clades resulting from duplication events than outgroup clades resulting from speciation events (Bottom) among all clades and by age class of the outer speciation event, measured in Ks units estimated at the nearest speciation preceding the duplication event. Asymmetry measures are Z2 for Ka; absolute normalized difference for |dPolarity| and EX. Negative t-values in Ka and |dPolarity| and positive values for the EX mean faster/more radical evolution in paralogs than in orthologous singletons. The same signs for the asymmetry indicate that the divergence between two paralogous branches is greater than the average of the divergence between their orthologous singleton and each of them. 2-tail P values are reported. [file 1745-6150-9-2-S3.doc]

Additional file 3: Table S1. Matched pairs two-tailed t-test of the hypotheses of relaxed constraint in paralogs than in singletons (Top) and higher asymmetry of evolution of clades resulting from duplication events than outgroup clades resulting from speciation events (Bottom) among all clades and by age class of the outer speciation event, measured in K_s_ units estimated at the nearest speciation preceding the duplication event. Asymmetry measures are Z^2^ for K_a_; absolute normalized difference for |dPolarity| and EX. Negative t-values in K_a_ and |dPolarity| and positive values for the EX mean faster/more radical evolution in paralogs than in orthologous singletons. The same signs for the asymmetry indicate that the divergence between two paralogous branches is greater than the average of the divergence between their orthologous singleton and each of them. 2-tail P values are reported.

| Clade age class, K_s_ | K_a_ | | |dPolarity| | | EX | |
| --- | --- | --- | --- | --- | --- | --- |
|  | T | P | t | P | t | p |
| Rates and radicality of changes | | | | | | |
| Overall | -19.32 | 8E-75 | -8.80 | 4E-18 | 7.83 | 9E-15 |
| 0 - 0.1 | -5.31 | 2E-07 | -2.54 | 0.0116 | 2.05 | 0.042 |
| 0.25 - 0.45 | -6.90 | 3E-11 | -3.08 | 0.0023 | 2.67 | 0.008 |
| 0.5 - 0.7 | -17.60 | 1E-60 | -10.56 | 8.5E-25 | 9.25 | 1.3E-20 |
| Asymmetry of changes | | | | | | |
| Overall | -4.65 | 4E-6 | 2.53 | 0.012 | -2.78 | 0.0054 |
| 0 - 0.1 | -1.96 | 0.051 | 3.01 | 0.003 | -3.22 | 0.0014 |
| 0.25 - 0.45 | -2.23 | 0.026 | 0.15 | >0.88 | -1.17 | >0.24 |
| 0.5 - 0.7 | -3.64 | 0.0003 | 0.68 | >0.50 | 0.47 | >0.64 |
