# Supplementary material for: Faster evolving Drosophila paralogs lose expression rate and ubiquity and accumulate more non-synonymous SNPs
Source: Biol Direct. 2014 Jan 17;9:2. doi: 10.1186/1745-6150-9-2 (PMC3906896; doi:10.1186/1745-6150-9-2)
Supplement: Additional file 4: Table S2 — Pairs of D. melanogaster paralogs demonstrating a significant rate asymmetry (false discovery rate adjustment (Benjamini & Yekutieli, 2001) at q<0.1). Gene family IDs correspond to those on http://www.indiana.edu/~hahnlab/fly/DfamDB/drosophila_frb.html. Pairwise columns shown: r – coefficient of correlation of expression level over 26 tissues; dME – signed difference in log mean expression level polarized by the differences in Ka; dCVE – signed difference in coefficient of variation of expression level in 26 tissues polarized by the differences in Ka; dKa/Ks_poly – signed difference in SNPs Ka/Ks polarized by the differences in divergence Ka. [file 1745-6150-9-2-S4.doc]

Additional file 4: Table S2. Pairs of *D. melanogaster* paralogs demonstrating a significant rate asymmetry (false discovery rate adjustment (Benjamini & Yekutieli, 2001) at q<0.1). Gene family IDs correspond to those on <http://www.indiana.edu/~hahnlab/fly/DfamDB/drosophila_frb.html>. Pairwise columns shown: r – coefficient of correlation of expression level over 26 tissues; dME – signed difference in log mean expression level polarized by the differences in K_a_; dCVE – signed difference in coefficient of variation of expression level in 26 tissues polarized by the differences in K_a_; dKa/Ks_poly – signed difference in SNPs Ka/Ks polarized by the differences in divergence K_a_.

| Gene family | Slower paralog | | | Faster paralog(s) | | | Pairwise data | | | | | | |
| --- | --- | --- | --- | --- | --- | --- | --- | --- | --- | --- | --- | --- | --- |
|  | ID | Name | K_a_ | ID | Name | K_a_ | Log_10_Z^2^ | r | dME | dCVE | dKa/Ks_poly | Molecular function | Relative location |
| 923 | FBgn0031170 | CG1718 | 0.154 | FBgn0034493 | CG8908 | 0.599 | 2.64 | -0.09 | -1.11 | -1.04 | 0.01 | transporters, enzymes | different arms |
|  |  |  |  | FBgn0261997 | CG42815 | 0.578 | 2.6 | -0.12 | -1.49 | -0.97 | 0.18 |  | different arms |
|  |  |  |  | FBgn0261998 | CG42816 | 0.578 | 2.6 | -0.12 | -1.49 | -0.97 | 0.18 |  | different arms |
|  |  |  |  | FBgn0036747 | CG6052 | 0.431 | 2.28 | -0.05 | -1.74 | -0.49 | 0.03 |  | different arms |
|  | FBgn0036747 | CG6052 | 0.431 | FBgn0034493 | CG8908 | 0.599 | 1.76 | 0.55 | 0.63 | -0.55 | -0.03 |  | different arms |
|  |  |  |  | FBgn0261998 | CG42816 | 0.578 | 1.65 | 0.16 | 0.25 | -0.48 | 0.15 |  | same arm nontandem |
|  |  |  |  | FBgn0261997 | CG42815 | 0.578 | 1.65 | 0.16 | 0.25 | -0.48 | 0.15 |  | same arm nontandem |
| 9357 | FBgn0040777 | CG14767 | 0.002 | FBgn0050357 | CG30357 | 0.851 | 2.61 | -0.28 | -2.06 | 2.24 | 0.09 | transporter/unknown | tandem collinear |
| 5717 | FBgn0036389 | short spindle 2 | 0.241 | FBgn0263232 | Nuclear export factor 3 | 0.802 | 2.56 | 0.53 | -0.82 | -0.15 | 0.14 | unknown | tandem collinear |
| 11408 | FBgn0030362 | regucalcin | 0.084 | FBgn0038258 | CG7362 | 0.838 | 2.5 | 0.01 | -2.32 | 1.07 | 0.22 | unknown or other /enzyme | different arms |
|  | FBgn0038257 | Senescence marker -30 | 0.267 |  |  |  | 2.26 | -0.11 | -1.98 | 0.5 | 0.24 |  | tandem collinear |
| 12880 | FBgn0032863 | Cell division cycle 23 ortholog | 0.034 | FBgn0051687 | CG31687 | 0.43 | 2.46 | 0.28 | -0.36 | 0.05 | 0.92 | enzyme/unknown | tandem collinear |
| 1504 | FBgn0025628 | CG4199 | 0.18 | FBgn0029596 | CG14054 | 0.847 | 2.38 | -0.16 | -1.64 | 2.92 | 0.26 | enzymes | tandem collinear |
|  | FBgn0032754 | CG10700 | 0.393 | FBgn0029596 | CG14054 | 0.847 | 2.03 | 0.57 | 0.57 | 2.89 | 0.09 |  | different arms |
|  | FBgn0025628 | CG4199 | 0.18 | FBgn0032754 | CG10700 | 0.393 | 1.64 | -0.11 | -2.21 | 0.04 | 0.18 |  | different arms |
| 4546 | FBgn0262519 | Mi-2 | 0.029 | FBgn0023395 | Chd3 | 0.277 | 2.31 | 0.59 | -0.92 | 1.92 | 0.19 | enzymes | same arm nontandem |
| 2260 | FBgn0037203 | slimfast | 0.112 | FBgn0036764 | CG5535 | 0.468 | 2.26 | 0.02 | -0.29 | -0.02 | 0.27 | transporters | same arm nontandem |
| 1532 | FBgn0027500 | spindle defective 2 | 0.017 | FBgn0036646 | CR18217 | 0.211 | 2.23 | 0.26 | 0.13 | -1.86 | . | unknown | different arms |
| 494 | FBgn0031738 | CG9171 | 0.076 | FBgn0032457 | CG15483 | 0.495 | 2.23 | -0.18 | -1 | 2.93 | 0.07 | enzymes | same arm nontandem |
| 604 | FBgn0003328 | scab | 0.16 | FBgn0034005 | Integrins: alphaPS4 subunit | 0.413 | 2.19 | 0.11 | -1.62 | -0.35 | 0.26 | other | tandem collinear |
|  |  |  |  | FBgn0034880 | alphaPS5 subunit | 0.392 | 2.12 | -0.03 | -1.57 | 0.37 | 0.3 |  | same arm nontandem |
| 9001 | FBgn0031998 | CG8451 | 0.085 | FBgn0053124 | CG33124 | 0.332 | 2.02 | 0.01 | -1.67 | -0.01 | 0.27 | transporters | same arm nontandem |
|  |  |  |  | FBgn0051262 | CG31262 | 0.319 | 1.97 | -0.15 | -1.39 | 1.43 | 0.11 |  | different arms |
| 8235 | FBgn0014029 | Septin 2 | 0.017 | FBgn0026361 | Septin 5 | 0.26 | 2.01 | 0.25 | -1.4 | 0.53 | 0.38 | enzymes | different arms |
| 9814 | FBgn0031771 | CG9140 | 0.016 | FBgn0034251 | CG11423 | 0.233 | 2 | -0.35 | -1.91 | 3.35 | 0.13 | enzymes | different arms |
| 10723 | FBgn0026874 | CG13358 | 0.384 | FBgn0025616 | CG13359 | 0.83 | 1.96 | -0.2 | 0.32 | 3.3 | 0.37 | unknown | tandem collinear |
| 11014 | FBgn0039273 | CG9996 | 0.245 | FBgn0054027 | CG34027 | 0.629 | 1.94 | -0.17 | -0.89 | 0.31 | 0.08 | unknown/enzyme | same arm nontandem |
| 5692 | FBgn0032264 | Lipase 4 | 0.104 | FBgn0032265 | CG18301 | 0.381 | 1.94 | 0.66 | -1.67 | -0.55 | 0.06 | enzymes | tandem collinear |
| 3937 | FBgn0030066 | CG1885 | 0.154 | FBgn0038361 | CG9589 | 0.535 | 1.9 | 0.48 | -0.04 | 3.92 | 0.01 | enzymes | different arms |
| 2144 | FBgn0035152 | CG3386 | 0.349 | FBgn0035151 | CG17129 | 0.787 | 1.9 | -0.15 | -0.4 | 1.14 | 0.53 | unknown | tandem collinear |
| 9191 | FBgn0015737 | Hemomucin | 0.127 | FBgn0041780 | Strictosidine synthase-like 2 | 0.4 | 1.89 | -0.09 | -1.99 | 0.13 | 0.17 | enzymes | same arm nontandem |
| 2757 | FBgn0051371 | CG31371 | 0.431 | FBgn0051014 | prolyl-4-hydroxylase-alpha SG1 | 0.725 | 1.89 | 0.11 | 1.17 | 2.13 | -0.08 | enzymes | tandem collinear |
| 5420 | FBgn0030314 | CG11696 | 0.257 | FBgn0030316 | CG11695 | 0.521 | 1.88 | 0.4 | -0.11 | -0.02 | -0.03 | nucleic acid binding | tandem collinear |
| 1453 | FBgn0003023 | ovarian tumor | 0.246 | FBgn0031622 | CG3251 | 0.525 | 1.87 | -0.05 | -0.2 | -0.45 | 0.1 | unknown | different arms |
| 903 | FBgn0010497 | Dietary and metabolic glutamate transporter | 0.071 | FBgn0028513 | CG9254 | 0.263 | 1.79 | -0.15 | -0.79 | 2.67 | -0.02 | transporters | same arm nontandem |
| 9296 | FBgn0032242 | CG5355 | 0.149 | FBgn0032969 | CG2528 | 0.327 | 1.78 | 0.01 | -0.94 | 3.69 | 0.34 | enzymes | same arm nontandem |
| 3802 | FBgn0053108 | CG33108 | 0.25 | FBgn0051184 | LSm3 | 0.868 | 1.77 | -0.05 | 0.77 | 0.27 | -0.22 | nuclear acid binding/unknown | tandem collinear |
| 6581 | FBgn0053199 | CG33199 | 0.132 | FBgn0033356 | CG8229 | 0.688 | 1.63 | 0.87 | 0.1 | -0.2 | 0.32 | unknown | tandem collinear |
| 2967 | FBgn0034842 | Proteasome beta5 subunit-related 1 | 0.133 | FBgn0051742 | Proteasome beta5 subunit-related 2 | 0.368 | 1.61 | 0.99 | -0.53 | -0.77 | 0.15 | enzymes | different arms |
| 6691 | FBgn0052181 | CG32181 | 0.597 | FBgn0052182 | CG32182 | 0.839 | 1.59 | -0.08 | -1.27 | -3.49 | -0.08 | unknown | tandem collinear |
| 10373 | FBgn0026314 | UDP-glycosyltransferase 35b | 0.236 | FBgn0040255 | Ugt86De | 0.417 | 1.58 | 0.87 | -1.93 | -1.01 | 0.49 |  | tandem collinear |
|  |  |  |  | FBgn0051002 | CG31002 | 0.417 | 1.57 | 0.08 | -1.89 | -1.31 | 0.14 | enzymes | same arm nontandem |
|  |  |  |  | FBgn0040253 | Ugt86Dg | 0.41 | 1.53 | 0.16 | -1.82 | -1.42 | 0.22 |  | tandem collinear |
| 4187 | FBgn0052271 | CG32271 | 0.21 | FBgn0035070 | CG3650 | 0.464 | 1.53 | 0.01 | -0.45 | -0.43 | 0.21 | enzymes | different arms |
| 932 | FBgn0030051 | Serine Protease Immune Response Integrator | 0.337 | FBgn0038113 | CG11668 | 0.557 | 1.5 | 0.09 | -0.36 | -1.46 | 0.11 | enzymes | different arms |
| 1604 | FBgn0050350 | CG30350 | 0.274 | FBgn0029809 | CG15767 | 0.47 | 1.43 | 1 | -0.8 | -0.7 | -0.04 | enzymes | different arms |
| 2468 | FBgn0037222 | CG14642 | 0.317 | FBgn0038114 | CG11670 | 0.498 | 1.35 | 0.2 | -1.24 | 2.18 | -0.15 | enzymes | same arm nontandem |
| 3776 | FBgn0050072 | Odorant-binding protein 50c | 0.506 | FBgn0050073 | Odorant-binding protein 50b | 0.726 | 1.19 | 0.97 | -0.26 | -1.46 | 0.03 | receptors | tandem collinear |
| 8563 | FBgn0028896 | CG17329 | 0.568 | FBgn0036421 | CG13481 | 0.788 | 1.15 | 1 | 0.04 | -0.38 | -0.46 | other | different arms |
|  | FBgn0051807 | CG31807 | 0.595 |  |  |  | 1.05 | 1 | 0.26 | 0.04 | . |  | different arms |
